# Joint modelling of longitudinal processes and time-to-event outcomes in heart failure: systematic review and exemplar examining the relationship between serum digoxin levels and mortality

**DOI:** 10.1186/s12874-023-01918-4

**Published:** 2023-04-19

**Authors:** Ryan J. Field, Carly Adamson, Pardeep Jhund, Jim Lewsey

**Affiliations:** 1 Health Economics and Health Technology Assessment, School of Health and Wellbeing, University of Glasgow, 90 Byres Road, Glasgow, G12 8TB UK; 2 School of Cardiovascular and Metabolic Health, University of Glasgow, Glasgow, UK

**Keywords:** Joint Modelling, Heart failure, Shared parameter models, Systematic review, Digoxin, Mortality

## Abstract

**Background:**

Joint modelling combines two or more statistical models to reduce bias and increase efficiency. As the use of joint modelling increases it is important to understand how and why it is being applied to heart failure research.

**Methods:**

A systematic review of major medical databases of studies which used joint modelling within heart failure alongside an exemplar; joint modelling repeat measurements of serum digoxin with all-cause mortality using data from the Effect of Digoxin on Mortality and Morbidity in Patients with Heart Failure (DIG) trial.

**Results:**

Overall, 28 studies were included that used joint models, 25 (89%) used data from cohort studies, the remaining 3 (11%) using data from clinical trials. 21 (75%) of the studies used biomarkers and the remaining studies used imaging parameters and functional parameters. The exemplar findings show that a per unit increase of square root serum digoxin is associated with the hazard of all-cause mortality increasing by 1.77 (1.34–2.33) times when adjusting for clinically relevant covariates.

**Conclusion:**

Recently, there has been a rise in publications of joint modelling being applied to heart failure. Where appropriate, joint models should be preferred over traditional models allowing for the inclusion of repeated measures while accounting for the biological nature of biomarkers and measurement error.

**Supplementary Information:**

The online version contains supplementary material available at 10.1186/s12874-023-01918-4.

## Background

Heart failure is a condition where there are well documented inter-relationships between numerous physical, biochemical and imaging characteristics and outcomes. Many studies tend to examine these associations with outcomes using data from one point in time such as randomization in a trial or the start of a cohort study. This fails to account for changes in characteristics over time. Just as baseline values may be associated with outcomes, changes in variables are also associated with changes in outcomes e.g., falling levels of natriuretic peptides are associated with lower mortality. However, analysing the association between changes in variables and outcomes is often performed with traditional time to event models using change values and starting follow up for outcomes once change has occurred. More recently joint modelling, combining two or more statistical models to increase efficiency and reduce bias, has gained favour in the literature as a method of dealing with this issue. The most common type of joint modelling within medicine is the joint modelling of repeat measure longitudinal data (e.g., repeated measures of biomarkers over time) and time-to-event data (i.e., survival data) which are linked through an association structure via shared random effects [1–4]. This seeks to improve efficiency and reduce bias in respect of treatment effect, censoring and mortality when compared against traditional models. Joint models (JMs) of this type are formed of two sub models: a longitudinal model such as a linear mixed effect (LME) model (which allows the modelling of longitudinal changes in biomarkers or other characteristics like blood pressure) and a survival model such as a Cox Proportional Hazards (Cox PH) model to model the outcome e.g., mortality. The LME model allows for both fixed and random effects accounting for non-independence of repeated measures from the same patient, whilst also allowing for unevenly spaced measurement occasions, biological variances, and measurement error [5]. The survival model allows for covariates and typically includes an association parameter representing the association between the longitudinal and survival process [2, 3, 5–7]. JMs which use data from randomized controlled trials (RCTs) can also model the overall treatment effect as well as the treatment effect on both the longitudinal and survival models [3] i.e., the effect on the characteristic and the effect on the outcome. This is analogous to other JMs which are used in the cardiovascular literature, for example in recurrent events analyses where models that examine the effect of a treatment on recurrent hospitalisations while also estimating a treatment effect for a terminal event such as death [8].

Given the increasing use of JMs, the aim of this paper is to review the application of joint modelling in heart failure and to provide guidance on how to assess and interpret results of joint modelling. To achieve this, we conduct a systematic review to identify and critically review current applications of joint modelling within the heart failure population and then present a critical summary of how joint modelling can be applied to heart failure data sets with use of an illustrative example. We examine the association between changes in serum digoxin levels and mortality in the Effect of Digoxin on Mortality and Morbidity in Patients with Heart Failure (DIG) trial as prior studies have tried to examine the association between digoxin levels and outcomes, and suggested that higher levels at one month following randomization may be associated with higher mortality [9].

## Methods

### Systematic review: joint modelling applications within heart failure

Our systematic review was conducted following the Preferred Reporting Items for Systematic Reviews (PRISMA) framework [10] and the protocol is registered with PROSPERO, registration number: CRD42020210056. The aim of the review was to identify journal articles which employed joint modelling on an adult heart failure population to review how joint modelling was being applied to heart failure.

#### Searches

Our search strategy is provided in the figure S1, Additional file 1. Medline, Embase, Scopus and Google Scholar were searched, with the last search being conducted on 10^th^ December 2021.

#### Screening

Articles were screened by two reviewers and full text was accessed for relevant articles. To capture all available articles no date limit was set and only English language articles were included. Only full text journal articles where joint modelling was applied to an adult heart failure population were considered for inclusion. Data were extracted by two reviewers.

### Exemplar: joint modelling of serum digoxin concentration and all-cause mortality

To demonstrate applications of JMs on heart failure data, the ‘The Effect of Digoxin on Mortality and Morbidity in Patients with Heart Failure’ (DIG) [11] trial was used. The dataset was obtained from the Biologic Specimen and Data Repository Information and Coordinating Center (BIOLINCC) under application #9257.

#### Statistical methods

Only data from patients on the treatment arm with at least one measurement of serum digoxin concentration (SDC) was used. SDC measurements were right skewed and therefore a square root (sqrt) transformation was applied. For this illustrate example, only patients with no missing covariates were included.

The *JM* Package was used to fit all joint models, this package allows the fitting of joint models of longitudinal and time-to-event data in R under a maximum likelihood approach [12].

Time must be modelled on the same scale for both models, and was modelled in the form of months (28 day calendar month) since randomisation; for SDC, time was taken as the specimen time. While the *JM* package allows for non-linear effects of time; for simplicity and ease of interpretation only linear terms were included.

The *JM* package requires an LME as fitted by the ‘*LME*’ function from the *nlme* package for the longitudinal sub-model [12]. For this example, both an unadjusted and adjusted LME model were fitted. With all models using sqrt SDC as the response variable. The unadjusted model included random intercepts as random effects. The adjusted model included the main effects of: estimated Glomerular Filtration Rate (eGFR), patient reported self-adherence, hours since last dose of the study drug and dose as fixed effects and included random intercepts and slopes for random effects. Full model equations for the LMEs and all other models are included in Table S1, Additional file 1.

Time-to-Event models for the JM package can be fit using either the ‘*coxph*’ or ‘*survreg*’ functions from the *Survival* package. For simplicity, Cox PH models fitted by the ‘*coxph*’ function were used. Like the LME both an unadjusted and adjusted model were fitted. The adjusted model containing the covariates of age, sex, ejection fraction, New York Heart Association class, history of hypertension, ischemic etiology of heart failure and body mass index. These covariates were selected on the basis of clinical relevance and prior knowledge of factors associated with outcomes in heart failure. The outcome examined was all-cause mortality.

Three JMs were constructed from both the unadjusted and adjusted LMEs and Cox PH Models as previously defined. Table 1 summarises the formulation of the JMs.

**Table 1:** Formulation of JMs Included in Exemplar

JM	LME	Cox PH	Time dependent parameter	Time dependent slope parameter
1	Unadjusted	Unadjusted	Y	N
2	Adjusted^a^	Adjusted^b^	Y	N
3	Adjusted^a^	Adjusted^b^	N	Y
4	Adjusted^a^	Adjusted^b^	Y	Y

*Legend*: ^a^Adjusted for Estimated Glomerular Filtration Rate (eGFR), patient reported self-adherence, hours since last dose of the study drug and dose

^b^Adjusted for age, sex, ejection fraction, New York Heart Association (NYHA) class, history of hypertension, ischemic etiology of heart failure and Body Mass Index (BMI)

As an additional analysis the JMs were compared against traditional models. The traditional models were Cox PH models using first and last measurements of sqrt SDC as a covariate and an extended Cox PH model including sqrt SDC as a time varying covariate. All models were adjusted for the same clinical covariates as the adjusted time-to-event models from the JMs. The model fit of the JMs were compared against each other using the Akaike Information Criterion (AIC). Likewise, the model fit of the traditional models were compared against each other using AIC. All models were compared for performance using a discrimination index: c-index for the traditional models and a dynamic discrimination index for the JMs. The dynamic discrimination index was obtained using the function ‘dynCJM’ from the JM package. Based on the time dependant discrimination index proposed by Antolini et al. the dynamic discrimination index in this context provides a single statistic to summarise the discrimination power of the model over the follow-up time and is calculated from a weighted average of time-dependant AUCs which is comparable to the well-known c-index. Like the c-index it does not take into account censoring [13, 14]. The parameter estimates and standard errors from the model were also compared. One hundred bootstrap samples were used to internally validate the comparison of the discrimination index.

For descriptive purposes, categorical variables are represented as percentages, continuous variables are represented as median (IQR). JM association parameters are represented as hazard ratios (HRs) and 95% confidence intervals (CI). A time dependent association parameter is the hazard of all-cause mortality per one unit increase of sqrt SDC at any time point. A time dependent slope association parameter is the hazard of all-cause mortality per one standard deviation increase in the slope of sqrt SDC at a time point (known as the instantaneous or current slope). A *p*-value of less than 0.05 is considered statistically significant.

All statistical analysis was conducted using R Version 4.0 [15] and *JM* package version 1.4–8 [12].

Ethical approval was not required for this systematic review and exemplar. All methods were carried out in accordance with relevant guidelines and regulations.

## Results

### Systematic review: use of joint modelling in heart failure

**Fig. 1:**
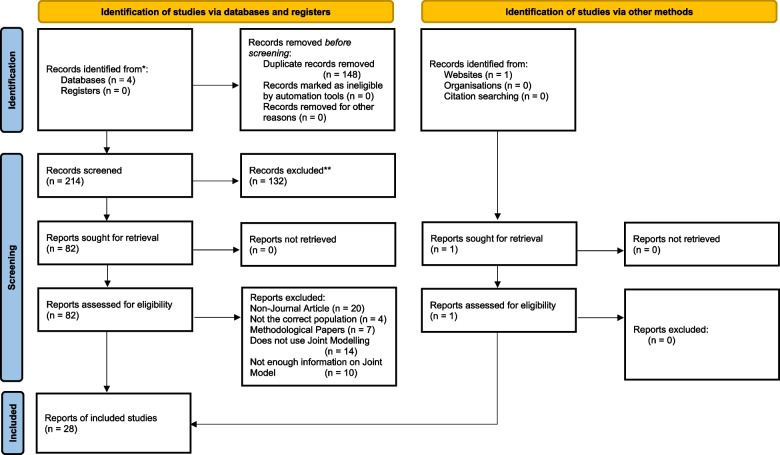
Prisma 2020 flow chart

Figure 1 shows the PRISMA flow diagram, with 28 studies meeting the criteria for inclusion. Table S2, Additional file 1 outlines the data sources of the 28 studies which met the inclusion criteria, the earliest included study being published in 2014 and between 4–7 studies being published per year from 2017 to the last search (10^th^ December 2021).

From the included studies, 25 (89%) used data from cohort studies and the remaining 3 (11%) studies used data from clinical trials. It is worth noting that 10 (36%) of the cohort studies used data from the Bio SHiFT study [16], likely because of the study design with its focus on repeated measurements of biomarkers.

From the studies, 22 (78%) exclusively included patients with heart failure, 6 of which specified patients with heart failure with reduced ejection fraction (HFrEF). The remaining 6 (22%) studies exclusively included patients with acutely decompensated heart failure. There were also studies which further selected patients on specific characteristics such as patients implanted with cardiac devices such as Cardioverter-Defibrillators (ICD) or Cardiac Resynchronization Therapy Devices (CRT-D), patients with advanced heart failure and patients who had undergone transcatheter mitral-valve repair.

#### Rationale

The most common rationale for using joint modelling was to assess the association of a biomarker with the hazard of an event. Other rationale included: joint modelling as a sensitivity analysis, reduction of bias due to censoring / mortality, comparison of prognostic models e.g., Weibull survival models and JMs, personalised prognostication using JMs, accounting for measurement error, different follow-up times and efficiency through combining data (i.e., smaller standard errors [5, 17]).

#### Longitudinal data

Table 2 summarises the longitudinal data used in the included studies; 21 (75%) studies used biomarkers with the most common biomarker being N-Terminal Pro-Brain Natriuretic Peptide (NT-proBNP). Some studies included multiple biomarkers in longitudinal sub-models, and some used multiple JMs of different biomarkers. The remaining studies used imaging parameters such as Left Ventricular Ejection Fraction (LVEF) and functional parameters such as health status, physical activity and depression for their longitudinal data. All but two studies specified their longitudinal sub-models as a linear mixed effects model.

**Table 2:** Summary of Longitudinal Data of Included Studies

Paper	Primary Longitudinal Data Type	Longitudinal Data	List of Biomarkers
Abebaw et al., 2021 [18]	Biomarker	Pulse Rate	Pulse Rate
Alvarez-Alvarez et al., 2021 [19]	Imaging Parameters	Left Ventricular Ejection Fraction	
Arnold et al., 2019 [20]	Functional Parameters	Health Status: KCCQ-OS	
Belay et al., 2021 [21]	Biomarker	Pulse Rate	Pulse Rate
Biegus et al., 2019 [22]	Biomarker	Meld-XI	Creatinine, Bilirubin
Bouwens et al., 2019 [23]	Biomarker	Cardiac Remodelling Biomarkers	ST2, Gal-3, Gal-4, GDF-15, MMP-2, MMP-3, MMP-9, TIMP-4, PLC, AP-N, CASP3, CTSD, CTSZ, CSTB, NT-ProBNP
Bouwens et al., 2020 [24]	Biomarker	Cell Adhesion Circulation Biomarkers	SELP, SELE, CDH5, ICAM-2, PECAM-1, C1qR, CHI3L1, CNTN1, EPHB4, Ep-CAM, ITGB2, JAM-A
Bouwens et al., 2020 [25]	Biomarker	Multiple Biomarkers	CCL15, CC16, CCL24, CXCL16, FAS, IL-1RT1, IL-1RT2, IL-17RA, IL-18BP, IL2-RA, IL-6RA, LTBR, TNF-R1, TNF-R2, TNFRSF10C, TNFRSF14, TNFSF13B
Brankovic et al., 2017 [26]	Biomarker	Renal Markers	Creatinine, eGFR, CysC, KIM-1, NAG, NAGL
Canepa et al., 2020 [27]	Biomarker	Multiple Biomarkers	SBP, Heart Rate, Haemoglobin, Creatinine, Uric Acid
Castelvecchio et al., 2018 [28]	Biomarker	Natriuretic Peptides	NT-ProBNP
Freedland et al., 2021 [29]	Functional Parameters	Depression: PHQ-9	
Hurst et al., 2019 [30]	Biomarker	Serum Lactate Dehydrogenase	LDH
Kelly et al., 2020 [31]	Functional Parameters	Physical Activity reported by ICD or CRT-D (Accelerometer Measurement of > 25 mg)	
Klimczak-Tomaniak et al., 2020 [32]	Biomarker	Macrophage and Neutrophil Related Biomarkers	M130(CD163), TRAP, GRN, SPON1, PGLYRP1, TFPI
Liu et al., 2018 [33]	Biomarker	Growth-Differentiation Factor	GDF-15
Nunez et al., 2014 [17]	Biomarker	Red Blood Cell Distribution Width	RDW
Nunez et al., 2017 [34]	Biomarker	Carbohydrate Antigen, Natriuretic Peptide	NT-ProBNP, CA125
Schreuder et al., 2021 [35]	Biomarker	Multiple Biomarkers	NT-ProBNP, HsTNT, CRP, Creatinine, eGFR, CysC, NAG, KIM-1
van Boven et al., 2017 [36]	Biomarker	MicroRNAs	miR-1254, miR-22-3p, miR-423-5p, miR-486-5p, miR-320a, miR-345-5p, miR-378a-3p
van Boven et al., 2018 [37]	Biomarker	Multiple Biomarkers	NT-ProBNP, HsTNT, CRP
van den Berg et al., 2019 [38]	Biomarker	Fibrinolysis Factors	PAI-1, tPA, uPA, suPAR
van den Berg et al., 2019 [39]	Imaging Parameters	Echocardiographic Parameters	
van den Berge et al., 2021 [40]	Imaging Parameters	Remodelling Parameters: LVEF, LVED, LVES	
van Vark et al., 2017 [41]	Biomarker	Galectin-3	Gal-3
van Vark et al., 2017 [42]	Biomarker	ST2	ST2
Veen et al., 2021 [43]	Imaging Parameters	Tricuspid regurgitation	
Zhang et al., 2018 [44]	Biomarker	Natriuretic Peptides	NT-ProBNP

*Legend*: *AP-N* aminopeptidase-N, *CASP3* caspase-3, *CSTB* cystatin-B, *CTSD* cathepsin D, *CTSZ* cathepsin Z, *eGFR* estimated glomerular filtration rate, *Gal-3* galectin-3, *Gal-4* galectin-4, *GDF-15* growth differentiation factor 15, *HsTnT* highly sensitive cardia45c troponin T, *MMP-2, 3, and 9* matrix metalloproteinase 2, 3, and 9, *NT-proBNP* N-terminal pro–B-type natriuretic peptide, *PLC* perlecan, *ST2* suppression of tumorigenicity-2, *TIMP-4* tissue inhibitor metalloproteinase 4, *C1qR Complement component* C1q receptor, *CDH5* Cadherin 5, *CHI3L1* Chitinase-3-like protein 1, *CNTN1* Contactin-1, *Ep-CAM* Epithelial cell adhesion molecule, *EPHB4* Ephrin type-B receptor 4, *ICAM-2 Intercellular adhesion* molecule-2, *ITGB2* Integrin beta-2, *JAM-A* Junctional adhesion molecule A, *PECAM-1* Platelet endothelial cell adhesion molecule 1, *SELE* E-selectin, *SELP* P-selectin, *CCL15* C–C motif chemokine 15, *CCL16* C–C motif chemokine 16, *CCL24* C–C motif chemokine 24, *CXCL16* C-X-C motif chemokine 16, *FAS* tumour necrosis factor receptor superfamily member 6, *IL-18BP* interleukin-18-binding protein, *IL-17RA* interleukin-17 receptor A, *IL2-RA* interleukin-2 receptor subunit alpha, *IL-6RA* interleukin-6 receptor subunit alpha, *IL-1RT1* interleukin-1 receptor type 1, *IL-1RT2* interleukin-1 receptor type 2, *LTBR* lymphotoxin b receptor, *TNF-R1* tumour necrosis factor receptor 1, *TNF-R2* tumour necrosis factor receptor 2, *TNFRSF14* tumour necrosis factor receptor superfamily member 14, *TNFRSF10C* tumour necrosis factor receptor superfamily member 10C, *TNFSF13B* tumour necrosis factor ligand superfamily member 13B, *CysC* cystatin C, *eGFR* estimated glomerular filtration rate, *NAG* N-acetyl-beta-D-glucosaminidase, *KIM-1* kidney injury molecule, *NGAL* plasma and urinary neutrophil gelatinase-associated lipocalin, *SBP* Systolic Blood Pressure, *NT-ProBNP* N-terminal pro-B-type natriuretic peptide, *PHQ-9* Patient Health Questionnaire-9, *CD163 (M130)* scavenger receptor cysteine-rich type 1 protein M130, *TRAP* tartrate-resistant acid phosphatase type 5, *GRN* granulins, *SPON1* spondin-1, *PGLYRP1* peptidoglycan recognition protein 1, *TFPI* tissue factor pathway inhibitor, *GDF-15* Growth-differentiation factor-15, *RDW* Red Blood Cell Distribution Width, *CA125* Carbohydrate Antigen 125, *miRs* microRNAs, *CRP* C-reactive protein, *PAI-1* plasminogen activator inhibitor 1, *tPA* tissue-type plasminogen activator, *uPA* urokinase-type plasminogen activator, *suPAR* soluble urokinase plasminogen activator surface receptor, *LVEF* Left ventricular ejection fraction, *LVED* left ventricular end-diastolic diameter, *LVES* left ventricular end-systolic diameter

#### Time-to-Event (Survival data)

Many studies included multiple events for their survival data through use of a composite outcome or multiple JMs. Table 3 shows that composite outcomes were the most common, but the events of composite outcomes varied by patient population as shown in Table S3, Additional file 1. The second most common event was all-cause mortality. Most models utilised Cox PH models for their survival sub-models with only two studies specifying a parametric Weibull model.

**Table 3:** Survival End Points of Included Studies

Endpoint	Overall (*N* = 41)
Endpoint	
All-Cause Mortality	12 (29.3%)
Cardiovascular Mortality	4 (9.8%)
Components of composite endpoint	2 (4.9%)
Components of the composite endpoint, MI, PCI, CABG, CVA and all-cause mortality	2 (4.9%)
Composite	17 (41.5%)
Default from Treatment	1 (2.4%)
Development of Anaemia	1 (2.4%)

*Legend*: Some studies included multiple JMs with different end points, so the total number of endpoints (41) is more than the number of included studies (27)

#### Missing data

Common joint modelling packages such as *JM* and *JMBayes* allow for both uneven spacing and missing longitudinal measurements. Both these packages require all covariates from both longitudinal and survival sub-models to be complete. This common limitation resulted in 13 (46%) of the included studies using imputation methods to complete missing data.

#### JM

All included studies modelled their JMs with R. The two most common packages being *JM* and *JMBayes*, with 7 (25%) studies using the *JM* package and 10 (36%) using *JMBayes and* another 3 (10%) studies specified both packages. Three studies used custom code, one study used the *joineRML* package and the remaining 3 (10%) did not specify the package used.

The R Packages used show both use of frequentist and Bayesian analysis. While the frequentist method use the maximum likelihood approach and is more comparable to more traditional models the Bayesian approach typically relies on Markov chain Monte Carlo (MCMC) sampling algorithms and may improve analysis by using related historical information and allowing for more flexible estimation [45].

#### Presentation of results

Generally, the results from the JMs included the hazard ratio of the associated longitudinal outcome of interest on the time-to-event outcome; this was typically either the association of the value of the longitudinal outcome or the slope of the outcome on the time-to-event model.

**Fig. 2:**
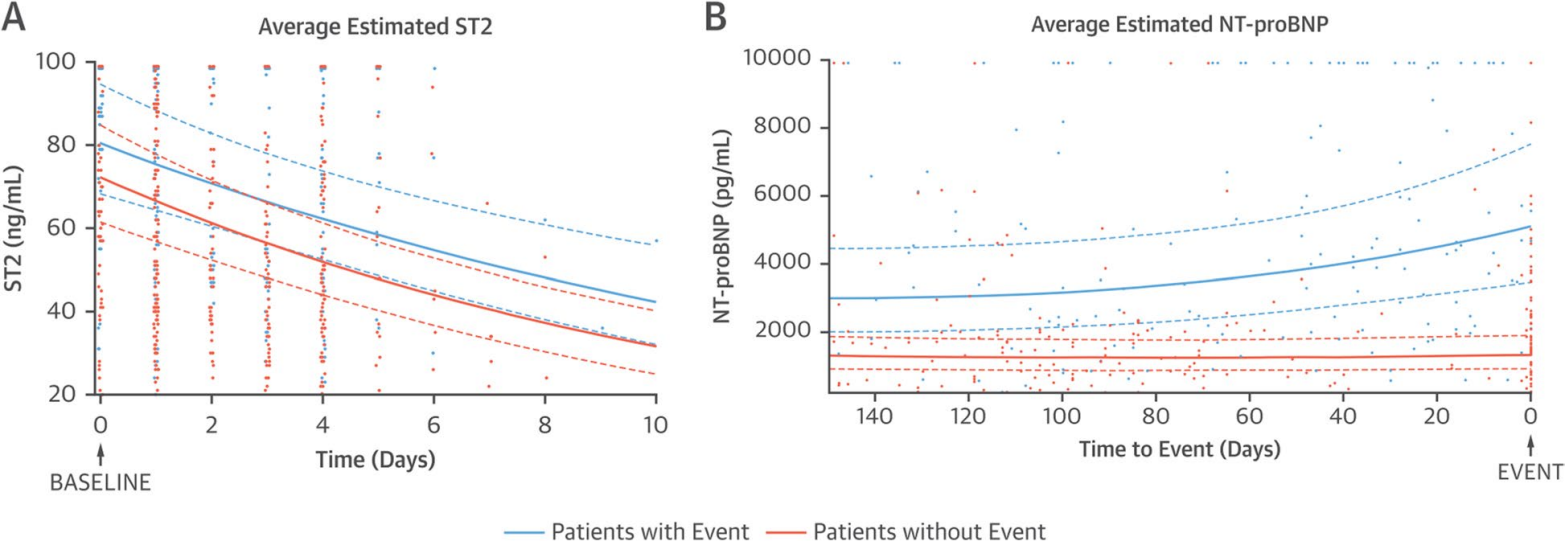
Average Estimated Biomarker Pattern, Combined with Individual Biomarker Measurements. During Follow-up in Patients with and without the Primary Endpoint from the van Vark et al. Study. Legend: Reprinted with permission from van Vark et al. © 2017 The American College of Cardiology Foundation [42]

The longitudinal sub-model is often presented as a coefficient or a graph of the average change in the longitudinal outcome of interest over time, these graphs are commonly split into groups of subjects e.g., those who did or did not experience the time-to-event outcome of interest. An example of this is illustrated in Fig. 2 from the Vark et al. study [42].

**Fig. 3:**
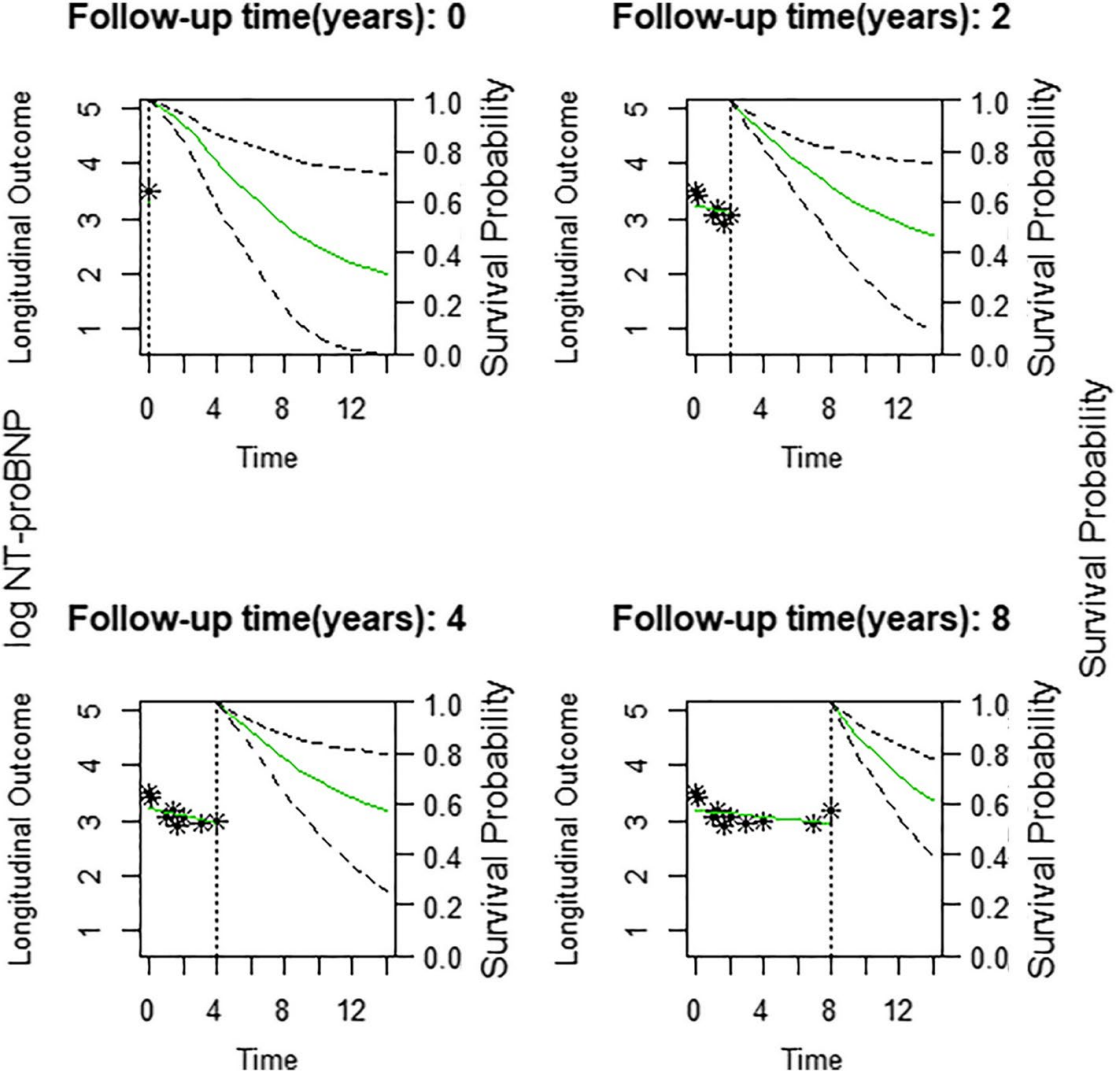
Dynamic survival probabilities with 95% CI based on various measurements of NT- ProBNP for a patient whose values fell. Legend: Reprinted with permission from Zhang et al. © 2018 Elsevier B.V. All rights reserved [44]

Commonly used packages in R provide capability to visually represent a trajectory of the longitudinal measure and the resulting changes in survival probability as shown in Fig. 3 taken from Zhang et al. where the trajectory of the longitudinal measures (NT-proBNP) is plotted on the left and the survival probability with 95% confidence intervals are plotted on the right. This is useful when looking at individual patient trajectories, in their example Zhang et al. show how the probability of survival changes in response to changes of the trajectory of NT-proBNP and a narrowing of the confidence intervals can be observed with the increase of measurements of NT-proBNP [44]. These such plots while useful were not common amongst the included studies.

Given this is a relatively new approach to analysing longitudinal data simultaneously with survival data studies often compare results from JMs against more traditional models such as a Cox PH model with only a singular measurement of the variable of interest.

#### Joint modelling outcomes

Generally, the JMs of the included studies performed favourably in terms of improving prognostication and identifying associations with adverse events. The Bio-SHiFT study being the most common data source explored a variety of biomarkers and imaging parameters including cell adhesion circulating biomarkers, fibrinolysis factors, renal markers, echocardiographic parameters, Micro Ribonucleic Acid (MiRNA’s), cardiac remodelling biomarkers and macrophage and neutrophil related biomarkers highlighting a variety of biomarkers and imaging parameters that were associated with the adverse events [23–26, 32, 35–39]. Whilst many of the tested biomarkers and parameters produced positive results, Van den berg et al. suggested that repeated measures of imaging parameters such as LVEF do not add any more value than single parameters due to the lack of change in those parameters over the observation period [39]. While this suggestion may be true for the population of the Bio-SHiFT study, both Alvarez-Alvarez et al. and Van den Berge suggested that for other heart failure populations repeated measurements of echocardiographic parameters such as LVEF can be useful, with Alvarez-Alvarez et al. investigating these parameters in a chronic heart failure population after CRT and Van den Berge exploring these parameters in an acute heart failure population [19, 40].

One of the key biomarkers which was explored in many studies was NT-ProBNP with Zhang et al., Castelvecchio et al. and Van Boven exploring its association with adverse events in a chronic heart failure population [28, 36, 44]. While these studies demonstrated the association between NT-ProBNP and adverse events, the Zhang et al. study suggested that the most recent value of NT-ProBNP had a similar predictive value as the serial measurements, but similar to the Van den Berg et al. study this may simply be due to the lack of change in values of NT-ProBNP within the study population and may not be generalisable [44].

Other key biomarkers which appeared in multiple studies were High sensitivity Troponin T (HsTnT), C-Reactive Protein (CRP), Cancer Antigen 125 (CA125), creatinine, Suppression of Tumorigenicity 2 (ST2), Galectin-3 (GAL-3) and Growth Differentiation Factor 15 (GDF-15) [22, 27, 33–35, 37, 41, 42], indicating that repeat measures of these markers are of interest within heart failure populations. Additionally, other less frequent markers included lactase dehydrogenase trends (LDH) [30], Red blood cell Distribution Width (RDW) [17] and ambulatory markers such as Systolic Blood Pressure (SBP), heart rate and haemoglobin [18, 21, 27].

Along with biomarkers, functional parameters were also of interest, with Kelly et al. taking a novel approach investigating the association of physical activity as reported by implanted devices i.e., by ICD or CRT-D [31] and Arnold et al. using the joint modelling of health status in the form of Kansas City Cardiomyopathy Questionnaire Overall Summary Score (KCCQ-OS) score and all-cause mortality as a sensitivity analysis to illustrate how censoring attenuated health status with respect to treatment effect [20].

Along with the echocardiographic parameters mentioned above imaging parameters were used in a total of four studies, using many of the parameters obtained from imaging such as LVEF, left ventricular end-diastolic diameter (LVED), left ventricular end-systolic diameter (LVES) and Tricuspid Regurgitation [40, 43].

### Association between serum digoxin concentration and mortality

#### Baseline characteristics

Table 4 shows the baseline characteristics of included patients (*n* = 2012), with a median age of 64 years, 22% of patients being women, median ejection fraction was 29% and 35% of patients died.

**Table 4:** Baseline Characteristics According to Patient Status (Dead / Censored) and Overall Sample (*n* = 2012)

	**Dead** **(** *n* **= 713)**	**Alive/ Censored** **(** *n* **= 1299)**	**Overall** **(** *n* **= 2012)**
**Age**			
Median (IQR)	66.0 (13.0)	63.0 (13.0)	64.0 (13.0)
**Sex**			
Female	151 (21.2%)	300 (23.1%)	451 (22.4%)
Male	562 (78.8%)	999 (76.9%)	1561 (77.6%)
**Ejection Fraction**			
Median (IQR)	25.0 (13.0)	30.0 (13.0)	29.0 (13.0)
**NYHA Class**			
Class I	75 (10.5%)	215 (16.6%)	290 (14.4%)
Class II	345 (48.4%)	741 (57.0%)	1086 (54.0%)
Class III	265 (37.2%)	325 (25.0%)	590 (29.3%)
Class IV	28 (3.9%)	18 (1.4%)	46 (2.3%)
**History of Hypertension**			
False	376 (52.7%)	727 (56.0%)	1103 (54.8%)
True	337 (47.3%)	572 (44.0%)	909 (45.2%)
**Ischemic HF**			
Ischemic	505 (70.8%)	939 (72.3%)	1444 (71.8%)
Non-ischemic	208 (29.2%)	360 (27.7%)	568 (28.2%)
**BMI**			
Median (IQR)	25.9 (5.91)	26.6 (5.97)	26.4 (5.76)

### JM – longitudinal data sqrt SDC over time

**Fig. 4:**
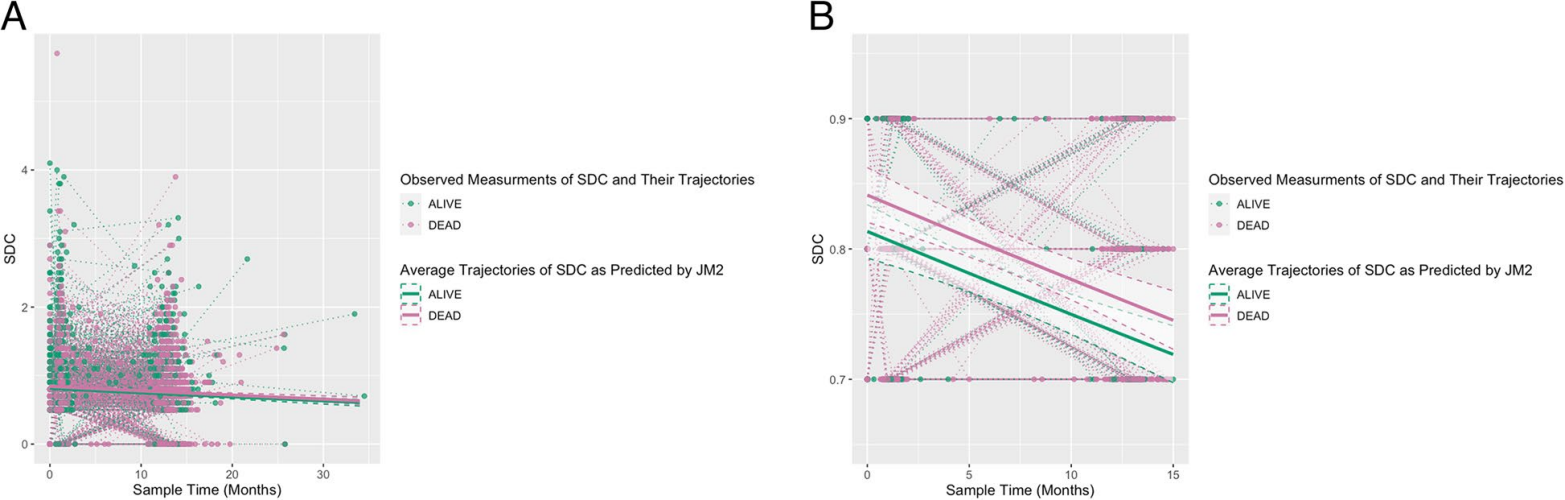
Average Trajectories of SDC by Patient Status as Predicted by JM 2 with Observed Values and Trajectories of SDC. Legend: **a** Average Trajectories of SDC by Patient Status as Predicted by JM 2 with Observed Values and Trajectories of SDC on original axis scale, **b** Average Trajectories of SDC by Patient Status as Predicted by JM 2 with Observed Values and Trajectories of SDC on scaled axis scaled for readability. Average Trajectories were predicted using JM2 for patients whose status were either alive or dead based on mean and mode characteristics (covariates) of each stratum of patients (Alive or Dead)

The coefficients from the longitudinal sub-model of JM 2 (Table S4, Additional file 1) for time -0.004 (-0.005—-0.002) suggests that the sqrt root SDC decreases by 0.004 per month after adjusting for covariates. Figure 4 shows a representation of the predicted, and therefore adjusted, average trajectories of SDC over time from JM 2, divided into patients who died during follow-up and those who did not, it suggests that patients who died had on average higher levels of SDC.

### JM – hazard ratios

Table 5 illustrates the results from the survival sub-models of the JMs in terms of a HR (95% CI) and *p*-value. All the JMs are time dependent relative risk models with a baseline risk function and as such the HRs can be interpreted similar to HRs from a proportional hazards model such as a Cox PH model. Focusing on the time dependant association parameter, the unadjusted model (JM 1) with a HR of 5.32 (3.07 – 9.22) suggesting a fivefold increase in the hazard of all-cause mortality per unit increase of sqrt SDC. This association is attenuated when adjusted for clinical covariates in JM 2 with a HR of 1.77 (1.34—2.33). The time dependent slope parameter of JM 3 is above the significance threshold (*p*-value 0.092) indicating insufficient evidence to establish an association between the slope of sqrt SDC and all-cause mortality. Neither the HR for the time dependant parameter or the HR for the time dependant slope parameter of JM are above the significance threshold (*p*-values of 0.427 and 0.13, respectively) suggesting insufficient evidence to establish an association with either value or slope when the model is adjusted for both. The interpretation of the time dependant parameter of this model would be the hazard of all-cause mortality per unit increase of SDC for patients having the same slope. The interpretation of the time dependant slope parameter of this model would be the hazard of all-cause mortality per one SD increase in slope for patients having the same level of sqrt SDC.

**Table 5:** Event Summary of JMs Represented Hazard Ratios

	**JM 1 (Unadjusted)**		**JM 2 (Adjusted)**		**JM 3** **(Adjusted Time Dependant Slopes)**		**JM 4** **(Time Dependant and Time Dependant slopes)**	
**Variable**	**HR**	***P*** ** value**	**HR**	***P*** ** value**	**HR**	***P*** ** value**		
Age			1.02 (1.01–1.03)	< 0.001	1.02 (1.01–1.03)	< 0.001	1.02 (1.01–1.03)	< 0.001
Male			1.19 (0.99–1.43)	0.062	1.2 (0.99–1.45)	0.064	1.22 (1.01–1.47)	0.042
Ejection Fraction %			0.97 (0.96–0.98)	< 0.001	0.97 (0.96–0.98)	< 0.001	0.97 (0.96–0.98)	< 0.001
NYHA Class II			1.22 (0.95–1.57)	0.115	1.2 (0.93–1.55)	0.171	1.22 (0.94–1.57)	0.131
NYHA Class III			1.66 (1.28–2.16)	< 0.001	1.63 (1.25–2.14)	< 0.001	1.66 (1.27–2.17)	< 0.001
NYHA Class IV			2.26 (1.45–3.53)	< 0.001	2.3 (1.43–3.72)	0.001	2.36 (1.48–3.76)	< 0.001
History of Hypotension			1.15 (0.99–1.34)	0.07	1.16 (0.99–1.36)	0.06	1.16 (0.99–1.35)	0.063
Non-Ischemic HF			1.07 (0.91–1.26)	0.428	1.07 (0.9–1.27)	0.455	1.08 (0.91–1.28)	0.386
BMI			0.98 (0.97–1)	0.036	0.98 (0.97–1)	0.061	0.99 (0.97–1)	0.113
Association (sqrt SDC)	5.32 (3.07–9.22)	< 0.001	1.77 (1.34–2.33)	< 0.001			1.33 (0.66–2.65)	0.427
Association Slope^a^					1.24 (0.97–1.59)	0.092	1.17 (0.96–1.42)	0.13

HR of all parameters except Association Slope reported as hazard of all-cause mortality per one unit increase at any point in time

^a^HR of Association Slope reported as hazard of all-cause mortality per one standard deviation increase in the slope of sqrt

### JM – individual patient trajectories

**Fig. 5:**
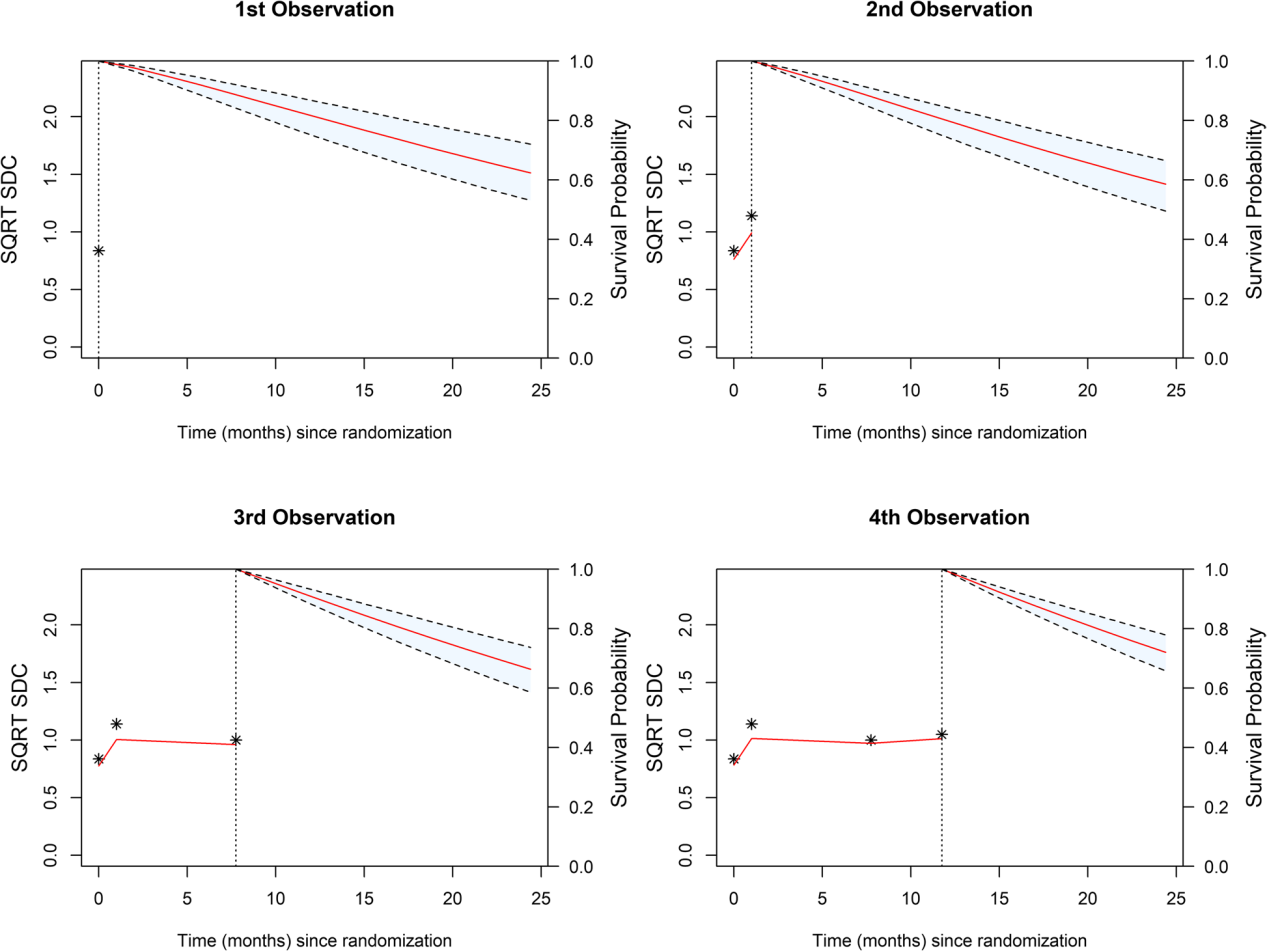
Individual Patient Trajectory of sqrt SDC and Survival Probability from Randomly Sampled Patient as Predicted by JM 2

Figure 5 shows the individual patient trajectories of a patient randomly selected (from patients with at least four measurements of sqrt SDC) at four different time points. These plots contain the longitudinal measurements of sqrt SDC as fitted by the JM on the left and the survival probability on the right, the dashed line indicating the last point the patient was known to be alive and the start of the survival curve, this point changes with each added measurement and as a result the survival curves are not directly comparable. However, these plots demonstrate how the measurements of sqrt SDC effects the survival probability and how the confidence intervals change over time with more measurements.

### Additional (Comparative) analysis

Results from the comparative analysis suggest that JM 2 had the overall best performance of the JMs with the lowest AIC, highest log likelihood and joint highest discrimination index of all models. The lower HR from the extended Cox PH model suggests that it underestimated the HR of the sqrt SDC parameter likely due to the nature of SDC as a time varying covariate; SDC being an endogenous biological covariate subject to measurement error, biological variances, being able to change between measurements and finally requiring the subject to be alive at measurement. Underestimation of the association parameter has been previously demonstrated in simulation studies [46].

Internal validation of the discrimination index using 100 bootstrap samples showed that JM2 with and mean discrimination index of 0.66 (range 0.6—0.72) outperformed the extended Cox PH model with a mean discrimination index of 0.65 (range 0.62 – 0.67) 71% of the time with respect to the discrimination index and 66% of the time when compared to Cox PH last measurement model with a mean discrimination index of 0.65 (range 0.62—0.68). The full additional analysis is available in Additional file 1.

## Discussion

In 2016, a systematic review by Sudell et al. showed an increase in use of JMs of longitudinal and time-to-event data over time. However, only 3 identified studies used ‘heart related’ data; the most common applications were to cancer and HIV/AIDS studies [47]. Developing on their search strategy.

We identified 28 studies by systematic review applying joint modelling within an adult heart failure population, and with use of an illustrative example have shown how to fit and interpret a JM. We have also shown how a JM approach can be used to examine the association between a biochemical test and outcomes in patients with heart failure.

Open-source software packages available in R such as *JM* and *JMBayes* make joint modelling more accessible reflected by the 20 (71%) studies using these packages. While these packages limit the JMs by way of underlying methodology [12, 48], if this methodology is not suitable custom code may be written as illustrated by the Hurst et al. study [30]. Both the JM and JMBayes packages also contain limitations around missing data in covariates, while the packages allow for missing longitudinal data, they do not allow for missing covariates in the sub models used to build the JMs, this results in the need to either use a complete case with regards to the covariates as shown in our exemplar or use imputation techniques such as multiple imputation as highlighted in the included studies.

Due the clinical nature of the included studies we found that studies often lacked details on the formulation of the JMs, e.g., the baseline risk function. Whilst this information could usually be derived by considering the packages used to fit the JMs, this information may be useful for reproducibility. We also identified that there was a lot of ambiguity around the origin of figures; whether or not they came from JMs or the individual components of the JMs e.g., a linear mixed effects model, modelled independently of the JM. We therefore suggest the need for clarity and transparency of the presentation of results from JMs.

It is also important that the results are easily understandable to a general audience. For example, the HR of a time dependent association parameter is intuitive but the HR of a time dependant slope parameter less so. Clinicians will often consider trends of biomarkers in day-to-day decision making so understanding these association parameters are key to relating them to clinical practice.

One driving motivation of the use of JMs was utilisation of repeated measures to inform prognosis and the comparison against a single measure. Most studies investigated biomarkers such as NT-ProBNP, CA125 and renal markers. However, JMs were not only limited to biomarkers; with such studies as van den Berg et al. investigating echocardiographic parameters [39], Arnold et al. focusing on health status [20] and Kelly et al. exploring physical activity as reported by an implanted device [31]. The use of these data illustrates the robustness of joint modelling. Another key rationale was the use of joint modelling to reduce bias due to censoring and mortality. Bias of this nature often occurs because subjects who are sicker are more likely to experience the event of interest or withdraw from the study earlier than those who are healthier leading to fewer longitudinal measurements [2]. To overcome this joint modelling provides a framework that acknowledges the underlying relationship between the longitudinal and event process through the use of a joint distribution [5]. The Arnold et al. study illustrates this bias visually highlighting how censoring likely attenuated heath status with respect to treatment effect [20]. Further, we have highlighted the use of joint models to handle missing not at random data through use of a joint distribution [5].

Only three studies used data from RCTs [20, 22, 27], as previously mentioned joint modelling can be used to reduce bias with respect to treatment effect. Whilst this highlights a potential gap in the literature it should be noted that during screening, we identified numerous studies using joint modelling as a sensitivity analysis with results consistent to those from separate longitudinal and survival models but were excluded from review as not enough details about the models were included for full appraisal.

Compared to cancer studies, there was a lack of focus on quality-of-life data with only one study including quality of life in the form of a Kansas City Cardiomyopathy Questionnaire and SF-36 scores [20], and one which included depression by means of patient health questionnaire 9 scores [29]. Whereas joint modelling with quality of life is much more prevalent in cancer studies [3, 20]. This highlights another area which may be of interest to future studies using joint modelling in heart failure.

The Bio-SHiFT Cohort made up 36% of studies primary data, illustrating how a study can be developed to fully use the capabilities of joint modelling; with frequent blood sampling and measurements of endpoints the study leads the way for further larger studies of this nature [16].

From the studies using data from the Bio-SHiFT Cohort we identified 3 studies which only selected baseline and the last two measurements closest to the endpoint [23, 38, 49]. While justified to investigate the trajectories before and after an event it should be noted that this kind of analysis could lead to bias and should only be conducted with proper justification.

Many of the included studies demonstrated how repeated measures added value with respect to both prognostication and model fit. The outcomes of the JMs illustrate how joint modelling can improve on traditional models and highlights the use of joint modelling to assess associations of various biomarkers, imaging parameters and functional parameters, and adverse outcomes as well as provide dynamic predictions. However, there were studies which stated repeat measurements did not add prognostic value or improve model fit. For example, Van den Berg et al. stated that repeated measurements of echocardiographic parameters were associated with adverse events but did not add prognostic value due to the lack of change in measurements over time [39]. Whist this may be true for the Bio-SHiFT cohort, both van den Berge et al. and Alvarez-Alvarez et al. illustrated that given the right context these repeat measurements can still add value [19, 40]. This highlights an important caveat regarding JMs, in that the cost may not outweigh the benefit of the JMs; whilst biomarkers are routinely collected at little added cost other parameters may be costly to collect and an understanding of the temporal patterns of these parameters prior to joint modelling is advisable.

Our exemplar shows how joint modelling can be applied to older studies in order to maximise information from data that was sometimes collected but unused in expensive clinical trials. We highlighted how they compare to traditional models and how they can compete and improve on these models while also providing new clinical insight. The HR of the extended Cox PH model when compared to JM2 and the last measurement Cox PH Model suggests that it underestimated the association parameter, as previously stated likely due to the nature of SDC as a covariate. Whilst the extended Cox PH allows for repeated measures it does not account for measurement error, biological variance or that SDC may vary between time points or after the last observed measurement; this underestimation has previously been demonstrated in in simulation studies [46]. Joint modelling while allowing for repeated measurements of SDC can handle the biological endogenous nature of SDC providing better inferences [5]. Our results suggest that higher values of SDC rather than the slope is associated with higher mortality in patients with heart failure. Our work extends the findings of prior studies that have tried to examine this association with landmark methods which do not perform as well as shown by our exemplar analysis. The implications of this finding are that for patients, their SDC level should be kept as low as possible while still maintaining adequate dosing and in patients with high SDC consideration may have to be given to reducing the dose. There is however an issue of reverse causality, sicker patients may be prescribed higher doses and consequently have higher SDC. However, higher SDC could still act as an indicator of risk and should alert clinicians to reassess the patient and consider other therapies for their heart failure.

Our exemplar only included patients who had no missing covariate values. While this is satisfactory for an exemplar, it can lead to loss of information and possible bias in research studies and the best practice may be to use multiple imputation [50]. However, it should be noted that multiple imputation requires pooling for valid inference, which may cause issues with computational complexity and the need for pooling for dynamic predictions.

Our internal validation using 100 bootstrap samples showed JM2 outperformed the extended Cox PH and Cox PH last measurement models with respect to the discrimination index most of the time (71% and 65% respectively) within the bootstrap samples providing validation to the prognostic performance of the JM. However, the range of the discrimination indices of JM2 is wider than both other models suggesting more variability of discrimination with the joint model within the bootstrap samples. While our exemplar used a dynamic discrimination index for prognostic comparison against traditional models, we found that there was little consistency in methods used to compare JMs against each other and traditional models, highlighting a need for consistency when evaluating JMs. We would suggest that any model specifications or parameters are clearly described to allow any comparisons to be made in future research.

JM 3 in our exemplar included a slope parameter corresponding to the rate of change in sqrt SDC at a time point, known as the instantaneous or current slope. As previously stated, this parameter can be difficult to interpret and as such the *JMBayes2* package offers the use of other slope parameters such as delta change i.e., change in the last month / year prior to the time point. This parameter should be easier to interpret and is likely to be more prognostic than currently used slope parameters [51].

Both our review and exemplar highlight the various output and figures that can be produced from a JM and show how powerful joint modelling can be, with applications for prognostication, research of the association of repeat measurements of biomarkers and an endpoint, sensitivity analysis and more.

While joint modelling has a variety of uses, it may be most beneficial in the presence of informative censoring or dropout, when incorporating time varying exogenous covariates such as biomarkers into survival models, and for prognostic modelling where dynamic predictions are useful. However joint modelling can be computationally complex and take longer to fit than traditional models. It should also be noted that inferences may only be valid where the joint model has been correctly specified both with respect to the sub models and baseline hazard function. Joint models are also only valid when conducted on the same population, this is to say that both the longitudinal and time-to-event responses need to come from the same group of subjects. Joint models may not provide better prognostic inference where there is limited variability in the repeated measures such as shown by van den Berg et al. [39].

Our exemplar has some limitations such as the limited number of repeat measurements. This may have affected the power to estimate the slope association parameter and overall accuracy of the model.

## Conclusions

In conclusion, this hybrid systematic review with exemplar highlights the rise in the use of JMs within heart failure, and our exemplar illustrates how JMs can be fitted to existing datasets adding value by utilising information from the repeated measures collected. This highlights why JMs are an increasingly popular alternative to traditional models such as Cox PH and Extended Cox PH.

## Supplementary Information

**Additional file 1: Supplementary Table S1.** Model equations for all models. **Supplementary Results.** Additional comparative analysis. **Supplementary Figure S1.** Full search strategy. **Supplementary Table S1.** Model equations for all models. **Supplementary Table S2.** Summary of included studies ordered by year. **Supplementary Table S3.** Breakdown of composite components of included studies. **Supplementary Table S4.** Coefficients from the longitudinal sub model from JM2. **Supplementary Table S5.** Performance summary of joint models, Cox PH and Extended Cox PH models. **Supplementary Table S6.** Hazard ratios and standard errors from cox models and JM2.

## Data Availability

The DIG study is available from the National Heart, Lung and Blood Institute: Biological Specimen and Data Repository Information and Coordinating Center (NIH BIOLINCC): https://biolincc.nhlbi.nih.gov/studies/dig/.

## Abbreviations

AIC: Akaike Information Criterion
BIOLINCC: Biologic Specimen and Data Repository Information and Coordinating Center
CA125: Cancer Antigen 125
CI: Confidence interval
Cox PH: Cox Proportional Hazards
CRP: C-Reactive Protein
CRT-D: Cardiac Resynchronization Therapy Devices
DIG: The Effect of Digoxin on Mortality and Morbidity in Patients with Heart Failure
eGFR: Estimated Glomerular Filtration Rate
GAL-3: Galectin-3
GDF-15: Growth Differentiation Factor 15
HR: Hazard Ratio
HFrEF: Heart Failure with reduced Ejection Fraction
HsTnT: High Sensitivity Troponin T
ICD: Cardioverter-Defibrillators
JM: Joint Model
KCCQ-OS: Kansas City Cardiomyopathy Questionnaire Overall Summary Score
LDH: Lactase Dehydrogenase
LME: Linear Mixed Effects
LVED: Left ventricular end-diastolic diameter
LVEF: Left Ventricular Ejection Fraction
LVES: Left ventricular end-systolic diameter
MCMC: Markov chain Monte Carlo
miRNA: Micro ribonucleic acid
NT-ProBNP: N-Terminal Pro-Brain Natriuretic Peptide
NYHA: New York Heart Association
PRISMA: Preferred Reporting Items for Systematic Reviews
RDW: Red blood cell Distribution Width
RCT: Randomized Control Trial
SBP: Systolic Blood Pressure
SDC: Serum Digoxin Concentration
Sqrt: Square Root
ST2: Suppression of Tumorigenicity 2
