# Supplementary material for: Joint modelling of longitudinal processes and time-to-event outcomes in heart failure: systematic review and exemplar examining the relationship between serum digoxin levels and mortality
Source: BMC Med Res Methodol. 2023 Apr 19;23:94. doi: 10.1186/s12874-023-01918-4 (PMC10114381; doi:10.1186/s12874-023-01918-4)
Supplement: Supplementary file 1 — Additional file 1: Supplementary Table S1. Model equations for all models. Supplementary Results. Additional comparative analysis. Supplementary Figure S1. Full search strategy. Supplementary Table S1. Model equations for all models. Supplementary Table S2. Summary of included studies ordered by year. Supplementary Table S3. Breakdown of composite components of included studies. Supplementary Table S4. Coefficients from the longitudinal sub model from JM2. Supplementary Table S5. Performance summary of joint models, Cox PH and Extended Cox PH models. Supplementary Table S6. Hazard ratios and standard errors from cox models and JM2. [file 12874_2023_1918_MOESM1_ESM.docx]

| Supplementary AppendixSupplementary Methods | |
| --- | --- |
| **Supplementary Table S1: Model Equations for All Models** | |
| **LME Models** | |
| Unadjusted LME | $\left\{ \begin{matrix} \sqrt{SDC}_{ij}= \beta_{0}+ \beta_{1}{Specimen Time}_{ij}+ b_{i0}+ \varepsilon_{ij}, \\ b_{i} \sim N\left( 0, D \right), \varepsilon_{ij} \sim N(0, \sigma^{2}) \end{matrix} \right.$ |
| Adjusted LME | $\left\{ \begin{matrix} \sqrt{SDC}_{ij}= \beta_{0}+ \beta_{1}{Specimen Time}_{ij}+ \beta_{2}{eGFR}_{ij}+\beta_{3}{Self Adherence}_{ij}+ \beta_{4}{Hours Since Last Dose}_{ij}+\beta_{5}{Dose}_{ij}+ b_{i0}+b_{i1}{Specimen Time}_{ij}+ \varepsilon_{ij}, \\ b_{i} \sim N\left( 0, D \right), \varepsilon_{ij} \sim N(0, \sigma^{2}) \end{matrix} \right.$ |
| **Cox PH Models** | |
| Unadjusted Cox PH | $h_{i}\left( t \right)=h_{0}\left( t \right)$ |
| Adjusted PH | $h_{i}\left( t \right)=h_{0}\left( t \right) exp(\gamma_{1}{Age}_{i}+ \gamma_{2}{Male}_{i}+ {\gamma_{3}Ejection Fraction}_{i}+ {\gamma_{4}NYHA Class II}_{i}+ {\gamma_{5}NYHA Class III}_{i}+ {\gamma_{6}NYHA Class IV}_{i}+ {\gamma_{7}History of Hypertension}_{i}+ {\gamma_{8}Non-Ischemic HF}_{i}+ {\gamma_{9}BMI}_{i}$) |
| **Joint Models** | |
| JM1 | $\left\{ \begin{aligned} y_{i}\left( t \right)= m_{i}\left( t \right)+ \varepsilon_{i \left( t \right)} \\ = \beta_{0}+ \beta_{1}Specimen Time+ b_{i0}+ \varepsilon_{ij} , \\ h_{i}\left( t \right)= h_{0}\left( t \right) exp\{\alpha m_{i}\left( t \right)\} \end{aligned} \right. \varepsilon_{i}\left( t \right)\sim N(0,\sigma^{2})$ |
| JM2 | $\left\{ \begin{aligned} y_{i}\left( t \right)= m_{i}\left( t \right)+ \varepsilon_{i \left( t \right)} \\ = \beta_{0}+ \beta_{1}Specimen Time+ \beta_{2}eGFR+\beta_{3}Self Adherence+\beta_{4}Hours Since Last Does+\beta_{5}Dose b_{i0}+b_{i1}t+ \varepsilon_{ij} , \varepsilon_{i}\left( t \right)\sim N\left( 0,\sigma^{2} \right), \\ \\ h_{i}\left( t \right)= h_{0}\left( t \right)\exp\left\{ \begin{aligned} \gamma_{1}{Age}_{i}+ \gamma_{2}{Male}_{i}+ {\gamma_{3}Ejection Fraction}_{i}+ {\gamma_{4}NYHA Class II}_{i}+ {\gamma_{5}NYHA Class III}_{i}+ \\ {\gamma_{6}NYHA Class IV}_{i}+ {\gamma_{7}History of Hypertension}_{i}+ {\gamma_{8}Non-Ischemic HF}_{i}+ {\gamma_{9}BMI}_{i}+\alpha m_{i}\left( t \right) \end{aligned} \right\}, \end{aligned} \right.$ |
| JM3 | $\left\{ \begin{aligned} y_{i}\left( t \right)= m_{i}\left( t \right)+ \varepsilon_{i \left( t \right)} \\ = \beta_{0}+ \beta_{1}Specimen Time+ \beta_{2}eGFR+\beta_{3}Self Adherence+\beta_{4}Hours Since Last Does+\beta_{5}Dose b_{i0}+b_{i1}t+ \varepsilon_{ij} , \varepsilon_{i}\left( t \right)\sim N\left( 0,\sigma^{2} \right), \\ \\ h_{i}\left( t \right)= h_{0}\left( t \right)\exp\left\{ \begin{aligned} \gamma_{1}{Age}_{i}+ \gamma_{2}{Male}_{i}+ {\gamma_{3}Ejection Fraction}_{i}+ {\gamma_{4}NYHA Class II}_{i}+ {\gamma_{5}NYHA Class III}_{i}+ \\ {\gamma_{6}NYHA Class IV}_{i}+ {\gamma_{7}History of Hypertension}_{i}+ {\gamma_{8}Non-Ischemic HF}_{i}+ {\gamma_{9}BMI}_{i}+\alpha m_{i}^{'}(t) \end{aligned} \right\}, \end{aligned} \right.$ |
| JM4 | $\left\{ \begin{aligned} y_{i}\left( t \right)= m_{i}\left( t \right)+ \varepsilon_{i \left( t \right)} \\ = \beta_{0}+ \beta_{1}Specimen Time+ \beta_{2}eGFR+\beta_{3}Self Adherence+\beta_{4}Hours Since Last Does+\beta_{5}Dose b_{i0}+b_{i1}t+ \varepsilon_{ij} , \varepsilon_{i}\left( t \right)\sim N\left( 0,\sigma^{2} \right), \\ \\ h_{i}\left( t \right)= h_{0}\left( t \right)\exp\left\{ \begin{aligned} \gamma_{1}{Age}_{i}+ \gamma_{2}{Male}_{i}+ {\gamma_{3}Ejection Fraction}_{i}+ {\gamma_{4}NYHA Class II}_{i}+ {\gamma_{5}NYHA Class III}_{i}+ \\ {\gamma_{6}NYHA Class IV}_{i}+ {\gamma_{7}History of Hypertension}_{i}+ {\gamma_{8}Non-Ischemic HF}_{i}+ {\gamma_{9}BMI}_{i}+\alpha_{1}m_{i}\left( t \right)+\alpha_{2}m_{i}^{'}(t) \end{aligned} \right\}, \end{aligned} \right.$ |
| **Comparative (Traditional) Models** | |
| Cox PH First Measurement | $h_{i}\left( t \right)=h_{0}\left( t \right) exp(\gamma_{1}{Age}_{i}+ \gamma_{2}{Male}_{i}+ {\gamma_{3}Ejection Fraction}_{i}+ {\gamma_{4}NYHA Class II}_{i}+ {\gamma_{5}NYHA Class III}_{i}+ {\gamma_{6}NYHA Class IV}_{i}+ {\gamma_{7}History of Hypertension}_{i}+ {\gamma_{8}Non-Ischemic HF}_{i}+ {\gamma_{9}BMI}_{i}+ \gamma_{10}{First Sqrt SDC}_{i}$) |
| Cox PH Last Measurement | $h_{i}\left( t \right)=h_{0}\left( t \right) exp(\gamma_{1}{Age}_{i}+ \gamma_{2}{Male}_{i}+ {\gamma_{3}Ejection Fraction}_{i}+ {\gamma_{4}NYHA Class II}_{i}+ {\gamma_{5}NYHA Class III}_{i}+ {\gamma_{6}NYHA Class IV}_{i}+ {\gamma_{7}History of Hypertension}_{i}+ {\gamma_{8}Non-Ischemic HF}_{i}+ {\gamma_{9}BMI}_{i}+ \gamma_{10}{Last Sqrt SDC}_{i}$) |
| Extended Cox PH | $h_{i}\left( t \right)=h_{0}\left( t \right) exp(\gamma_{1}{Age}_{i}+ \gamma_{2}{Male}_{i}+ {\gamma_{3}Ejection Fraction}_{i}+ {\gamma_{4}NYHA Class II}_{i}+ {\gamma_{5}NYHA Class III}_{i}+ {\gamma_{6}NYHA Class IV}_{i}+ {\gamma_{7}History of Hypertension}_{i}+ {\gamma_{8}Non-Ischemic HF}_{i}+ {\gamma_{9}BMI}_{i}+ \alpha{SQRT SDC}_{i}(t)$) |

## Supplementary Results

*Additional (Comparative) analysis*

Supplementary Table S5 shows the performance characteristics of the JMs and traditional models. JM 2 had the best overall performance, with the lowest AIC (7273.89) and highest log likelihood (-3613.94) of the JMs and joint highest discrimination index of all models. The extended Cox PH model performed better than the traditional models with the lowest AIC (10217.12) and highest log likelihood (-5188.38). JM 1 had the worse overall performance of the JMs with the highest AIC, lowest log likelihood and lowest discrimination index. The two traditional models performed similarly to each other, with equal log likelihood and marginal differences in AIC and the last measurement model having a slightly better discrimination index of (0.65). This discrimination index of 0.65 shared by JM 2, the last measurement and extended Cox PH models suggest that the last measurement of SDC has a similar prognostic performance to repeated measurements of SDC.

Supplementary Table S6 shows the hazard ratios from the Cox PH models and JM 2. It shows a marginal difference between the hazard ratio of Sqrt SDC from the last measurement model (1.78) and the time dependant association parameter JM 2 (1.77). Both the extended cox and first measurement model had lower hazard ratios for Sqrt SDC of 1.73 and 1.52 respectively. The extended cox model appears to have underestimated the HR of the sqrt SDC parameter, likely due to the nature of SDC as a covariate; SDC is a biological endogenous covariate, which is to say it has special properties that need to be considered and respected. These properties include: the need for the patient to be alive for the measurement, the measurement is subject to biological variances and measurement error, the measurement may only be observed at specific time points but may vary between these time points. This underestimation of the association parameter has previously been demonstrated in simulation studies such as those by Sweeting and Thompson [1]. Using one hundred bootstrap samples, the discrimination index of JM2, the Extended Cox PH and Cox PH last measurement models were compared to internally validated the results of the discrimination index. From the 100 bootstrap samples JM 2 outperformed the extended Cox PH Model 71% of the time with respect to the discrimination index and 66% of the time when compared to the Cox PH Last Measurement model. The discrimination indices from JM2 within the 100 bootstrap samples had a mean of 0.66, median of 0.66 and range of 0.6 – 0.72, whilst the extended cox PH model had a mean of 0.65, median of 0.65 and range of 0.62 – 0.67 and the cox PH last measurement model having a mean of 0.65, median of 0.65 and range of (0.62 – 0.68).

## Supplementary Figure S1. Full search strategy:

## Supplementary Table S2, Summary of Included Studies Ordered by Year

| **Paper** | **Year** | **Primary Data Source (Study)** | **Primary Study Type** | **Included Study Population Size** | **Type of HF** | **Specific Characteristics** |
| --- | --- | --- | --- | --- | --- | --- |
| Nunez et al., 2014 [3] | 2014 | Custom COHORT | COHORT | 1702 | Acute |  |
| Nunez et al., 2017 [4] | 2017 | Custom COHORT | COHORT | 946 | Acute |  |
| Brankovic et al., 2017 [5] | 2017 | Bio SHiFT | COHORT | 263 | Chronic |  |
| van Vark et al., 2017 [6] | 2017 | TRIUMPH | COHORT | 475 | Acute |  |
| van Vark et al., 2017 [7] | 2017 | TRIUMPH | COHORT | 475 | Acute |  |
| van Boven et al., 2017 [8] | 2017 | Bio SHiFT | COHORT | 263 | Chronic |  |
| Zhang et al., 2018 [9] | 2018 | Custom COHORT | COHORT | 1998 | Chronic |  |
| Castelvecchio et al., 2018 [10] | 2018 | Biomarker Plus | COHORT | 143 | Chronic | Ischemic |
| Liu et al., 2018 [11] | 2018 | Custom COHORT | COHORT | 173 | Chronic | Ischemic, HFrEF |
| van Boven et al., 2018 [12] | 2018 | Bio SHiFT | COHORT | 263 | Chronic |  |
| Hurst et al., 2019 [13] | 2019 | Custom COHORT | COHORT | 323 | Chronic | Advanced Heart Failure |
| Arnold et al., 2019 [14] | 2019 | COAPT | RCT | 611 | Chronic | HFrEF, Patients randomised to Transcatheter Mitral-Valve Repair vs standard therapy |
| Biegus et al., 2019 [15] | 2019 | RELAX-AHF | RCT | 1120 | Acute |  |
| van den Berg et al., 2019 [16] | 2019 | Bio SHiFT | COHORT | 263 | Chronic |  |
| van den Berg et al., 2019 [17] | 2019 | Bio SHiFT | COHORT | 106 | Chronic | HFrEF |
| Bouwens et al., 2019 [18] | 2019 | Bio SHiFT | COHORT | 263 | Chronic |  |
| Kelly et al., 2020 [19] | 2020 | Boston Scientific ALTITUDE registry | COHORT | 20927 | Chronic | With ICD / CRT-D |
| Bouwens et al., 2020 [20] | 2020 | Bio SHiFT | COHORT | 263 | Chronic |  |
| Bouwens et al., 2020 [21] | 2020 | Bio SHiFT | COHORT | 250 | Chronic |  |
| Klimczak-Tomaniak et al., 2020 [22] | 2020 | Bio SHiFT | COHORT | 263 | Chronic |  |
| Canepa et al., 2020 [23] | 2020 | GISSI-HF | RCT | 5469 | Chronic | HFrEF |
| Veen et al., 2021 [24] | 2021 | EUROMACS | COHORT | 2496 | Chronic | After LVAD Implantation |
| van den Berge et al., 2021 [16] | 2021 | Custom COHORT | COHORT | 111 | Acute | New Onset HF |
| Alvarez-Alvarez et al., 2021 [25] | 2021 | Custom COHORT | COHORT | 328 | Chronic | After Cardiac Resynchronization therapy |
| Schreuder et al., 2021 [26] | 2021 | Bio SHiFT | COHORT | 250 | Chronic |  |
| Abebaw et al., 2021 [27] | 2021 | Custom COHORT | COHORT | 302 | Chronic |  |
| Belay et al., 2021 [28] | 2021 | Custom COHORT | COHORT | 271 | Chronic |  |
| Freedland et al., 2021 [29] | 2021 | Custom COHORT | COHORT | 400 | Chronic |  |

HFrEF: Heart Failure Reduce Ejection Fraction, ICD: Implantable Cardioverter-Defibrillator, CRT-D: implantable cardiac resynchronization therapy (CRT) defibrillator, LVAD: left ventricular assist device.

## Supplementary Table S3, Breakdown of Composite Components of Included Studies

| **Composite Components** |
| --- |
| All-Cause Mortality and HF Hospitalisation |
| Cardiac Death, Heart Transplantation, Left Ventricular Assist Device Implantation and Hospitalisation for the Management of acute or Worsened HF |
| Suspected Pump Thrombosis and Confirmed Pump Thrombosis |
| LV assist device implantation, heart transplantation, or all-cause mortality |
| Cardiac Death, Cardiac Transplant, LVAD Implantation and Hospitalisation for the management of Acute or Worsened HF |
| All-Cause Mortality and Readmission for HF |
| Hospitalisation for the Management of Acute or Worsened Heart Failure, LVAD Implantation, Cardiac Transplantation and Cardiac Death |
| MACE: Major Adverse Cardiac Events (All Cause Death, MI, and First HF Rehospitalization) |

## Supplementary Table: S4, Coefficients from the Longitudinal Sub model from JM2

| **Variable** | **Value** | **Standard Error** | **p-Value** |
| --- | --- | --- | --- |
| Intercept | 0.9798 | 0.0153 | <0.0001 |
| Specimen Time (Months) | -0.0036 | 0.0006 | <0.000 |
| eGFR | -0.0049 | 0.0002 | <0.0001 |
| Self-Adherence: None | -0.0570 | 0.0122 | <0.0001 |
| Self-Adherence: Some | -0.1498 | 0.0221 | <0.0001 |
| Number of Hours Since Dose | -0.0016 | 0.0001 | <0.0001 |
| Dose | 1.1306 | 0.0503 | <0.0001 |

## Supplementary Table: S5, Title: Performance Summary of Joint Models, Cox PH and Extended Cox PH models.

| **Measure** | **Joint Model 1**  **(Basic)** | **Joint Model 2**  **(Adjusted)** | **Joint Model 3**  **(Adjusted**  **Time Dependent Slopes)** | **Joint Model 4 (Adjusted Time Dependant and Time Dependant Slopes)** | **Cox PH Model 1**  **(First Measurement)** | **Cox PH Model 2 (Last Measurement)** | **Cox PH Model 3 (Extended Cox)** |
| --- | --- | --- | --- | --- | --- | --- | --- |
| AIC | 10889.64 | 7273.89 | 10352.85 | 10355.42 | 10291.65 | 10277.86 | 10217.12 |
| Log Likelihood | -5437.82 | -3613.94 | -5153.43 | -5153.71 | -5220.84 | -5220.84 | -5188.38 |
| Discrimination Index | 0.56 | 0.65 | 0.65 | 0.65 | 0.64 | 0.65 | 0.65 |

The Discrimination Index for all joint models was calculated using *dynCJM* function from the JM package, for all Cox PH models c-index was used for Discrimination Index. Joint Models were fit using Maximum Likelihood to obtain AIC.

|  | **First Measurement Model** | | | **Last Measurement Model** | | | **Extended Cox Model** | | | **Joint Model 2** | | |
| --- | --- | --- | --- | --- | --- | --- | --- | --- | --- | --- | --- | --- |
| **Variable** | **HR** | **SE** | **p-value** | **HR** | **SE** | **p-value** | **HR** | **SE** | **p-value** | **HR** | **SE** | **p-value** |
| Age | 1.02 | 0.004 | <0.001 | 1.02 | 0.004 | <0.001 | 1.02 | 0.004 | <0.001 | 1.02 | 0.004 | <0.001 |
| Male | 1.18 | 0.094 | 0.079 | 1.19 | 0.094 | 0.06 | 1.18 | 0.094 | 0.079 | 1.19 | 0.094 | 0.062 |
| Ejection Fraction % | 0.97 | 0.004 | <0.001 | 0.97 | 0.004 | <0.001 | 0.97 | 0.004 | <0.001 | 0.97 | 0.004 | <0.001 |
| NYHA Class II | 1.21 | 0.128 | 0.14 | 1.21 | 0.128 | 0.138 | 1.20 | 0.128 | 0.157 | 1.22 | 0.128 | 0.115 |
| NYHA Class III | 1.65 | 0.134 | <0.001 | 1.63 | 0.134 | <0.001 | 1.62 | 0.134 | <0.001 | 1.66 | 0.134 | <0.001 |
| NYHA Class IV | 2.22 | 0.226 | <0.001 | 2.27 | 0.226 | <0.001 | 2.23 | 0.226 | <0.001 | 2.26 | 0.227 | <0.001 |
| History of Hypotension | 1.15 | 0.077 | 0.066 | 1.16 | 0.077 | 0.06 | 1.16 | 0.077 | 0.059 | 1.15 | 0.077 | 0.07 |
| Non-Ischemic HF | 1.07 | 0.085 | 0.456 | 1.06 | 0.085 | 0.499 | 1.05 | 0.085 | 0.529 | 1.07 | 0.085 | 0.428 |
| BMI | 0.98 | 0.008 | 0.033 | 0.98 | 0.008 | 0.033 | 0.98 | 0.008 | 0.038 | 0.98 | 0.008 | 0.036 |
| Sqrt SDC | 1.52 | 0.114 | <0.001 | 1.78 | 0.113 | <0.001 | 1.73 | 0.114 | <0.001 |  |  |  |
| Time dependent association parameter for sqrt SDC* |  |  |  |  |  |  |  |  |  | 1.77 | 0.140 | <0.001 |

## Supplementary Table: S6, Title: Hazard Ratios and Standard Errors from Cox Models and JM2

*The time dependent association parameter for sqrt SDC gives the hazard of all-cause mortality per 1 unit increase in square root transformed SDC at any time point. This HR can be compared with the HR for sqrt SDC from the Cox models.

# References for Additional File 1

[1] M. J. Sweeting and S. G. Thompson, “Joint modelling of longitudinal and time-to-event data with application to predicting abdominal aortic aneurysm growth and rupture,” *Biom J*, vol. 53, no. 5, pp. 750–763, Sep. 2011, doi: 10.1002/BIMJ.201100052.

[2] F. Hsieh, Y. K. Tseng, and J. L. Wang, “Joint Modeling of Survival and Longitudinal Data: Likelihood Approach Revisited,” *Biometrics*, vol. 62, no. 4, pp. 1037–1043, Dec. 2006, doi: 10.1111/J.1541-0420.2006.00570.X.

[3] J. Nunez *et al.*, “Red blood cell distribution width is longitudinally associated with mortality and anemia in heart failure patients,” *Circulation Journal*, vol. 78, no. 2, pp. 410–418, 2014, doi: http://dx.doi.org/10.1253/circj.CJ-13-0630.

[4] J. Nunez *et al.*, “Long-term serial kinetics of N-terminal pro B-type natriuretic peptide and carbohydrate antigen 125 for mortality risk prediction following acute heart failure,” *Eur Heart J Acute Cardiovasc Care*, vol. 6, no. 8, pp. 685–696, 2017, doi: http://dx.doi.org/10.1177/2048872616649757.

[5] M. Brankovic *et al.*, “Patient-specific evolution of renal function in chronic heart failure patients dynamically predicts clinical outcome in the Bio-SHiFT study”, doi: 10.1016/j.kint.2017.09.013.

[6] L. C. van Vark *et al.*, “Prognostic value of serial galectin-3 measurements in patients with acute heart failure,” *J Am Heart Assoc*, vol. 6, no. 12, 2017, doi: http://dx.doi.org/10.1161/JAHA.116.003700.

[7] L. C. van Vark *et al.*, “Prognostic Value of Serial ST2 Measurements in Patients With Acute Heart Failure,” *J Am Coll Cardiol*, vol. 70, no. 19, pp. 2378–2388, 2017, doi: http://dx.doi.org/10.1016/j.jacc.2017.09.026.

[8] N. van Boven *et al.*, “Serially measured circulating miR-22-3p is a biomarker for adverse clinical outcome in patients with chronic heart failure: The Bio-SHiFT study,” *Int J Cardiol*, vol. 235, pp. 124–132, 2017, doi: http://dx.doi.org/10.1016/j.ijcard.2017.02.078.

[9] J. Zhang, P. Pellicori, D. Pan, R. Dierckx, A. L. Clark, and ..., *Dynamic risk stratification using serial measurements of plasma concentrations of natriuretic peptides in patients with heart failure*. Elsevier, 2018. [Online]. Available: https://www.sciencedirect.com/science/article/pii/S0167527317374296

[10] S. Castelvecchio *et al.*, “Longitudinal profile of NT-proBNP levels in ischemic heart failure patients undergoing surgical ventricular reconstruction: The Biomarker Plus study,” *Int J Cardiol*, vol. 260, pp. 24–30, 2018, doi: http://dx.doi.org/10.1016/j.ijcard.2018.02.084.

[11] J. X. Liu *et al.*, “Repeated measurement of growth-differentiation factor-15 in Chinese Han patients with post-myocardial infarction chronic heart failure,” *Journal of Geriatric Cardiology*, vol. 15, no. 10, pp. 618–627, 2018, doi: http://dx.doi.org/10.11909/j.issn.1671-5411.2018.10.002.

[12] N. van Boven, L. C. Battes, K. M. Akkerhuis, and ..., *Toward personalized risk assessment in patients with chronic heart failure: detailed temporal patterns of NT-proBNP, troponin T, and CRP in the Bio-SHiFT …*. Elsevier, 2018. [Online]. Available: https://www.sciencedirect.com/science/article/pii/S0002870317303368

[13] T. E. Hurst *et al.*, “Dynamic prediction of left ventricular assist device pump thrombosis based on lactate dehydrogenase trends,” *ESC Heart Fail*, vol. 6, no. 5, pp. 1005–1014, 2019, doi: 10.1002/ehf2.12473.

[14] S. v Arnold *et al.*, “Health Status After Transcatheter Mitral-Valve Repair in Heart Failure and Secondary Mitral Regurgitation: COAPT Trial,” *J Am Coll Cardiol*, vol. 73, no. 17, pp. 2123–2132, 2019, doi: https://doi.org/10.1016/j.jacc.2019.02.010.

[15] J. Biegus, B. Demissei, D. Postmus, G. Cotter, and ..., “Hepatorenal dysfunction identifies high‐risk patients with acute heart failure: insights from the RELAX‐AHF trial,” *… Heart Failure*, 2019, [Online]. Available: https://onlinelibrary.wiley.com/doi/abs/10.1002/ehf2.12477

[16] J. C. van den Berge *et al.*, “Left ventricular remodelling and prognosis after discharge in new-onset acute heart failure with reduced ejection fraction,” 2021, doi: 10.1002/ehf2.13299.

[17] V. J. van den Berg *et al.*, “Repeated Echocardiograms Do Not Provide Incremental Prognostic Value to Single Echocardiographic Assessment in Minimally Symptomatic Patients with Chronic Heart Failure: Results of the Bio-SHiFT Study,” *Journal of the American Society of Echocardiography*, vol. 32, no. 8, pp. 1000–1009, 2019, doi: http://dx.doi.org/10.1016/j.echo.2019.04.419.

[18] V. J. van den Berg *et al.*, “Longitudinally Measured Fibrinolysis Factors are Strong Predictors of Clinical Outcome in Patients with Chronic Heart Failure: The Bio-SHiFT Study,” *Thromb Haemost*, vol. 119, no. 12, pp. 1947–1955, 2019, doi: http://dx.doi.org/10.1055/s-0039-1696973.

[19] J. P. Kelly *et al.*, “Association of Implantable Device Measured Physical Activity With Hospitalization for Heart Failure,” *JACC Heart Fail*, vol. 04, 2020, doi: http://dx.doi.org/10.1016/j.jchf.2019.10.009.

[20] E. Bouwens *et al.*, “Temporal patterns of 14 blood biomarker candidates of cardiac remodeling in relation to prognosis of patients with chronic heart failure-the Bio-SHiFT study,” *J Am Heart Assoc*, vol. 8, no. 4, 2019, doi: http://dx.doi.org/10.1161/JAHA.118.009555.

[21] E. Bouwens *et al.*, “Serially Measured Cytokines and Cytokine Receptors in Relation to Clinical Outcome in Patients With Stable Heart Failure,” *Canadian Journal of Cardiology*, vol. 36, pp. 1587–1591, 2020, doi: 10.1016/j.cjca.2020.08.010.

[22] D. Klimczak-Tomaniak *et al.*, “Temporal patterns of macrophage- and neutrophil-related markers are associated with clinical outcome in heart failure patients,” *ESC Heart Fail*, 2020, doi: http://dx.doi.org/10.1002/ehf2.12678.

[23] M. Canepa, G. Siri, M. Puntoni, R. Latini, L. Tavazzi, and A. P. Maggioni, “Testing longitudinal data for prognostication in ambulatory heart failure patients with reduced ejection fraction. A proof of principle from the GISSI-HF database,” *Int J Cardiol*, 2020, doi: http://dx.doi.org/10.1016/j.ijcard.2020.03.064.

[24] K. M. Veen *et al.*, “Clinical impact and ‘natural’ course of uncorrected tricuspid regurgitation after implantation of a left ventricular assist device: An analysis of the European Registry for Patients with Mechanical Circulatory Support (EUROMACS),” *European Journal of Cardio-thoracic Surgery*, vol. 59, no. 1, pp. 207–216, Jan. 2021, doi: 10.1093/ejcts/ezaa294.

[25] B. Alvarez-Alvarez *et al.*, “Long-term cardiac reverse remodeling after cardiac resynchronization therapy,” *J Arrhythm*, vol. 37, p. 653, 2021, doi: 10.1002/joa3.12527.

[26] M. M. Schreuder *et al.*, “Sex-specific temporal evolution of circulating biomarkers in patients with chronic heart failure with reduced ejection fraction,” 2021, doi: 10.1016/j.ijcard.2021.04.061.

[27] Y. Abebaw, K. Mohammed, A. Aragaw, and B. Melese, “Joint Modeling of Longitudinal Pulse Rate and Time-to-Default from Treatment of Congestive Heart Failure Patients,” *Research Reports in Clinical Cardiology*, vol. 12, pp. 41–52, Oct. 2021, doi: 10.2147/RRCC.S326229.

[28] A. T. Belay, D. B. Belay, S. G. Gebremichael, and S. B. Agegn, “Congestive Heart Failure Patients’ Pulse Rate Progression and Time to Death at Debre Tabor Referral Hospital, Ethiopia,” *Adv Public Health*, vol. 2021, pp. 1–8, Nov. 2021, doi: 10.1155/2021/9550628.

[29] K. E. Freedland, B. C. Steinmeyer, R. M. Carney, J. A. Skala, L. Chen, and M. W. Rich, “Depression and Hospital Readmissions in Patients with Heart Failure,” *American Journal of Cardiology*, vol. 0, no. 0, Dec. 2021, doi: 10.1016/J.AMJCARD.2021.10.024.
